# Real-time fake news detection in online social networks: FANDC Cloud-based system

**DOI:** 10.1038/s41598-024-76102-9

**Published:** 2024-10-29

**Authors:** Nadire Cavus, Murat Goksu, Bora Oktekin

**Affiliations:** 1 Department of Computer Information Systems, Near East University, 99138 Nicosia, Mersin 10, Cyprus Turkey; 2 Computer Information Systems Research and Technology Center, Near East University, 99138 Nicosia, Mersin 10, Cyprus Turkey

**Keywords:** Mathematics and computing, Computational science, Computer science, Information technology, Software

## Abstract

Social networks have become a common way for people to communicate with each other and share ideas, thanks to their fast information-sharing features. But fake news spread on social networks can cause many negative consequences by affecting people’s daily lives. However, the literature lacks online and real-time fake news detection systems. This study aims to fill this gap in the literature and to handle the fake news detection problem with a system called FANDC, based on cloud computing, to cope with fake news in seven different categories, and to solve the real-time fake news detection problems. The system was developed using the CRISP-DM methodology with a hybrid approach. BERT algorithm was used in the system running on the cloud to avoid possible cyber threats with the dataset created with approximately 99 million big data from COVID-19-TweetIDs GitHub repository. It was trained in two periods with 100% accuracy during the modeling phase in terms of training accuracy. Experimental results of the FANDC system performed the real-time detection of fake news at 99% accuracy. However, previous studies experimental level success rate in the literature, were around 90%. We hope that the developed system will greatly assist social network users in detecting fake news in real-time.

## Introduction

Over the last decade, the rapid growth of online social networks (OSNs) such as Twitter, Facebook, Instagram, and WhatsApp have weakened the importance of traditional print media while becoming an essential tool for sharing and disseminating daily news^1^. Accordingly, people’s ways of accessing daily news and information quickly shifted to OSNs with their socialization efforts. OSNs were initially platforms for sharing personal information and finding out who was where. Users could communicate online with people in the network whenever they wanted. However, these networks are used for communication and information gathering purposes as well as socialization^2^. In this way, OSNs have suddenly turned from a platform where personal situations are shared to a platform where some news is followed. Moreover, these OSNs have been platforms where users can get quick responses and feedback about where they live or on different global topics, where they can share their feelings, thoughts, and concerns^3^. There has been an increase in the use of OSN Especially with the COVID-19 pandemic which affected change in user’s daily life due to the rapid and dizzying developments in social, economic, political, and health fields. In today’s current information age, many social network users tend to disseminate news shared on OSNs for various purposes. Some of the disseminated news contents raises questions as some may contain false information believed to be accurate or malicious disinformation for propaganda purposes^4^. However, many methods, including machine learning-based (ML) and today’s artificial intelligence (AI) applications, have been developed to detect this fake news shared in OSNs^5^. It is of great importance that OSN users can make the necessary checks in real-time in case of any doubt. A system that they can control is needed to protect them from the harmful effects of the fake news they encounter on social networks. If the user shares the fake news they are exposed to, thinking it is true, it causes misinformation -that is, the unintentional spread of disinformation.

However, these studies did not proceed beyond experimental level. Prior studies were mostly carried out using ready-made datasets which are either labeled fake or real. A vast majority studies focused on the performance of ML and AI applications rather than detecting fake news and providing feedback to the OSN user. In the same vein, the use of big datasets were avoided by prior studies due to limited computational resources, hence, the use of smaller datasets. In addition, prior studies detected fake news using a single problem approach like propaganda, disinformation or satire rather than fake news detection by categorization. Furthermore, there are many features that distinguish the proposed FANDC fake news detection system in this study from these studies. The first and most important of these is in its creation of a system-specific corpus with use of big dataset. The second important feature is in the categorization evaluation of fake news into seven subcategories. These produce more enlightenment and meaningful results as a result of the feedback it gives to the OSN user. Another important feature is that it is a cloud-based computing system integrated with distributed and container structures which protect the system against possible cyber threats.

A system called Fake News Detection on Cloud (FANDC) has been developed to cope with this rapidly spreading fake news detection problem in OSNs and, at the same time, to fill this gap identified in the literature. The major contribution the system has brought to the literature is that it uses cloud computing, a vital structure against cyber threats, by using the Bidirectional Encoder Representations from Transformers (BERT) algorithm, which has proven its success in the Natural Language Processing (NLP) field. Another innovation is that, by leveraging state-of-the-art technology, fake news can be detected in seven subcategories within OSNs. This breakthrough ensures that misinformation is effectively monitored and prevented from causing harm. These are clickbait, disinformation, hoaxes, junk news, misinformation, propaganda, and satire. The focus of this study is not on detecting fake news with a general approach. Instead, it is to divide fake news into seven sub-categories and offer OSN users more information about the content. In this context, the system in question was designed based on cloud computing and considered more secure than providing services via a domain name over the internet. The primary purpose of this study is to develop an online real-time detection system based on cloud computing as a different approach to detecting fake news spread in OSNs^6^. The research questions created within this framework are presented below:

- Is it possible to detect fake news spread on OSNs online and in real-time with the developed system?
- With the developed system, is it possible to categorize fake news spread in OSNs?

The rest of the paper is phased as follows: In the “related works” section, previous studies in this field are summarized along with the gap in the literature. This is followed by the “methods” section, which explains in detail the CRISP-DM methodology used to create the FANDC system. Additionally, it explains the steps of obtaining and processing the data which included; data pre-processing, categorization, and the design which highlights the technical specifications and evaluation criteria of the proposed system. The “results” section which comes after shows the experimental results. In the “discussion” section, a comprehensive and comparative analysis on the obtained results are discussed. And finally, in the “conclusion” section, a summary of the model results performance evaluation alongside recommendations for future studies are explained.

### Related works

Most fake news detection systems use ML techniques to help users distinguish whether the news they view on OSNs is fake^7^. These systems compare the given data with some previously known corpus containing fake/false and correct information^8–10^. In addition to these studies, there are also studies that try to solve the fake news detection problem on the basis of emotions^11^ and approaches based on opposition news networks^12^. Although their success rates are high, they are not real-time and are only at the experimental level. According to a study conducted by Goksu and Cavus^13^, it is stated that a wide range of fake news detection studies were carried out using various ML and AI techniques. These studies were created by labeling both false/fake and accurate data in a pre-trained model^14^. In the study of Krishna and Adimoolam^15^, a system was presented by comparing logistic regression and support vector machines from ML algorithms. However, the focus here is on the comparison of algorithms. On the other hand, Della Vedova et al.^16^ created a system by combining news content and social context features on a previously labeled data set. In this study, a determination was made on the Facebook chatbot. The success rate achieved was 81.7%.

However, there are also classically created and human-centered fake news detection sites besides these techniques. For websites such as Teyit.org, Malumatfurus.org, and DogruluPayi.com in Turkey. News verification on these websites is done manually^17^. Experts use various criteria to verify news sources, including publication date, web page access, cross-verification, objective reading, accuracy questioning, official source verification, and validation on other platforms. As one might expect, manual verification or detection of fake news on the Internet on an individual scale is extremely slow, expensive, and subjective. It may contain inspector biases and is challenging to apply to large volumes of data. In this context, when the fake news detection ecosystem is examined, a structure consisting of the news source, its title, its content, and the image or video shared in the news content is generally encountered.

In their fake news studies, Mathews and Preeth^18^ adopted a content-based approach. Accordingly, the text to be analyzed focuses only on the content, and no further auxiliary elements are needed. Content-based methods are also examined in three parts: knowledge-based, language/style-based, and posture-based. Since fake news inherently contains elements such as misinformation or information pollution, the main purpose of detecting this is to check the accuracy of the claims in the content. So, being able to detect fake news on OSNs automatically is difficult. According to Beer and Methi^19^, detection is done in two ways: manually and automatically. Verification checking is usually done manually, on an informed basis, and by a team. Some systems automatically check for accuracy. Current validation approaches are expert-driven, crowdsourced, and computationally driven.

On the other hand, Unver^14^, examining expert-focused verification systems in his research, revealed that verifying or fact-checking sources that are thought to be fake or contain fake news requires a lot of human resources. Examples of these are websites from Turkey, such as Teyit.org, Dogrulukpayi.com, and Malumatfurus.org. In addition, the crowdsourced verification system of Allen et al.’s^20^ work was examined. The aim here is to present the accuracy of the news content to the information of the participants, to make an evaluation according to the result to be obtained, and thus to confirm generally accepted news based on the knowledge of society. Computational fact-checking often includes network-based semantic approaches. It deals with the criterion of semantic closeness between the concepts in the data set to be analyzed. The important thing in this analysis is to find the complexity of case control by finding the shortest path between concepts.

In Farinha and Carvalho’s study^21^, case controls using infographics checked whether the claims in the news content could be deduced from the existing facts in the infographic. Language-based approaches may be the best approach to detecting fake news in OSNs, but they are difficult to succeed when the focus shifts. Since bot accounts often use this linguistic approach in OSNs and, occasionally, official language usage may shift to another specific area, such methods make it very difficult to detect evolved styles. However, malicious fake news publishers use certain writing styles to impress large communities, spread distorted, misleading information, and build a specific audience.

In their study, Jiang and Wilson^22^ analyzed news content with a stylistic approach and tried to identify similarities and differences in writing style. Similarly, Mahyoob et al.^23^ revealed a study on a stylistic/language-based approach by detecting misleading statements and claims in the news content, where the objectivity in the news content decreased and turned into a more rhetorical form. The stance-based approach was examined in the study of Hossain^24^. Accordingly, misleading rhetorical language is generally used in news content. For instance, the news writer can ironically write about the pros and cons of coronavirus vaccines from his perspective. Different arguments can be made against other points of view using the misleading concepts of satire and rumor. In their studies, Shu et al.^25^ defined propagation/network-based fake news detection as generally focusing on users’ profiles but showing homogeneous and heterogeneous features.

Therefore, detecting the spread/network-based fake news spread by generating information (flood) from different networks is quite challenging as they are required to spread out a little indication between OSNs for them to be detected. However, in the case of in-homogeneous OSNs on Twitter, if they cannot be detected the first time, they will in the long run as they are easy to detect using several different fake news detection models. An example of fake news spread based on inhomogeneous propaganda networks is the use of bot accounts for the spread of Twitter posts (tweets) via retweets^26^. The detection of source-based fake news is one of the relatively detectable structures today that is due to the fact that they are spread in OSNs by certain groups for primarily political and ideological ideas which influences the minds of those who can related to it. For this reason, a hybrid detection method is seen as a more effective coping method which involves applying several fake news detection methods together^27,28^.

A summary of the recent studies, their methodologies and algorithms used, along with their advantages and disadvantages, are presented in Table 1. From the table, it has been shown that the use of a single method for detecting fake news will not be a problem mitigation approach. Also, the discussed methods above are not only time-consuming and subjective but they also do not meet end-user needs as there is always a need to be sure that the suspected fake news is easily detectable by the users. In addition, avoidance of cyber threats and provision of ubiquitous support to users is a necessity. In light of these data, it has been determined that the gap in the literature is that, online and real-time fake news detection in OSNs is not categorized.

**Table 1:** Summary of related works.

References	Methodology/Techniques	Algorithms	Advantageous	Disadvantageous
Khan et al.^8^	Comparison	BERT, Bi-LSTM, RoBERT	Focused on algorithm selection	Lack of application of the model to OSN data
Zhang et al.^9^	Comparison	Computing Cold-Start Ratio, Estimating Minimal Processing Time	Focused on the system success rate 97%	Lack of application of the model to OSN data
Dixit et al.^10^	Data mining	Hybrid CNN model optimized with a Levy flight honey badger algorithm	Focused on algorithms and the system success rate 97%	Lack of application of the model to OSN data
Dixit et al.^14^	Comparison	PPCA, LSTM-LF	Focused on system success rate 98%	Lack of application of the model to OSN data
Krishna & Adimoolam^15^	Comparison	LR-SVM	Focused on algorithms performance evaluation LR 95.12%, SVM 91.68%	There was no focus on fake news detection
Della Vedova et al.^16^	Content-based, Social-based	LR	This study focused on content	81.7% success rate
Mathews &Preethi^18^	ML	Doc2Vec, LR, SVM, KNN, PAC, RF, BEC, VEC	The algorithms performance evaluation success rate ranges between 95.8–96.7%	There was no focus on fake news detection
Allen et al.^20^	Comparison	LR-RF	The algorithms performance evaluation success rate ranges from 56–92%	There was no focus on fake news detection
Farinha & Charvalho^21^	NLP	Fuzzy, Syntax	Focused on system success	Despite high success rate of 90%
Jiang & Wilson^22^	Linguistic Model	SVM	Focused on system success, with rate between 76% and 87%	May be suitable for hybrid approach
Mahyoob et al.^23^	Linguistic Model	QDA	Focused on system success	Low success rate
Hossain^24^	NLP	BERT	Focused on system success	Low success rate
Shu et al.^25^	Data mining	ML Algorithms	Focused on dataset comparison and detection algorithm	Low success rate
Zhou & Zafarani^26^	Network-Based	SVM, KNN, NB, DT, RF	System success rate between 80% and 90%, early detection	Limited data set
Mu & Aletras^27^	Hybrid	SVM, Avg-EMB, BiGRU-ATT, ULMFiT, T-BERT, H-BERT, T-XLNet, H-XLNet	System success rate 79.7% and early detection	Limited data set
Xia et al.^28^	Hybrid	CNN-BiLSTM-AM	Focused on system success	Despite 98% highest success
This Work	CRISP-DM	BERT	99% high system success, big data	User requirement

## Methods

Perceiving fake news spread in OSNs as a business problem that needs to be tackled today, the CRISP-DM methodology, defined as the Cross-Industry Standard Process for Data Mining, has been adopted to solve this business problem. Within the framework of the CRISP-DM methodology, the first stage is having an understanding of the data, the processes of collecting the data, defining its properties, performing exploratory analyses, and evaluating the data quality were carried out. Eventually, poor quality, irrelevant data, or passing to the next stages with insufficient information about the existing data will adversely affect the process. Data volume (size, number of records, total databases, tables), data attributes and definitions (variables, data types), relationship and mapping schemes (understanding attribute representations), and basic descriptive statistics (mean, median, variance) were analyzed. Our main goal here is to do detailed research on the data and to understand the data. Data quality analysis is the part where we analyze the data quality in our datasets and resolve data-related issues before further analyzing the data or embarking on modeling work. This stage is the last in the data meaning stage, where we identify the errors, deficiencies, and problems that must be resolved. Data quality analysis focuses on missing values, inconsistent values, incorrect information due to data errors, and inaccurate metadata information.

The next stage in the CRISP-DM process is the data preparation stage, after obtaining sufficient information about the problem and the related data set. Data preparation refers to cleaning, modifying, organizing, and preparing data before running an analysis or ML model. This stage is the most time-consuming part of the text-mining process. This stage took 60–70% of the completion time of the study and identified that this percentage is suitable^29^. In this process, which was carried out meticulously on 99 million Twitter data from COVID-19-TweetIDs GitHub repository, the data was ready for the modeling phase. Otherwise, bad data, containing complex data without data preprocessing, leads to bad modeling, lousy performance, and bad results. In the data pre-processing phase, the punctuation marks, stop-words, deleting numbers, tokenization, stemming, lemmatization, etc., were applied first to bring the data to the desired criteria. At this stage, since tweets had to be categorized, synonym tagging was performed within the framework of concepts such as click-bait, disinformation, hoax, junk news, misinformation, propaganda, and satire, which are used within the framework of fake news. To collect data via Twitter and to use the functions of this application in another application like Python or .Net, a streaming API was requested from Twitter. With this streaming API, tweets posted on GitHub during the COVID-19 pandemic by Chen et al.^30^ (from COVID-19-TweetIDs GitHub repository) were downloaded within the framework of academic ethics. The study is limited to the data collected from Twitter, which is the OSN site used extensively during COVID-19.

### The proposed fake news detection system on cloud computing

**Fig. 1:**
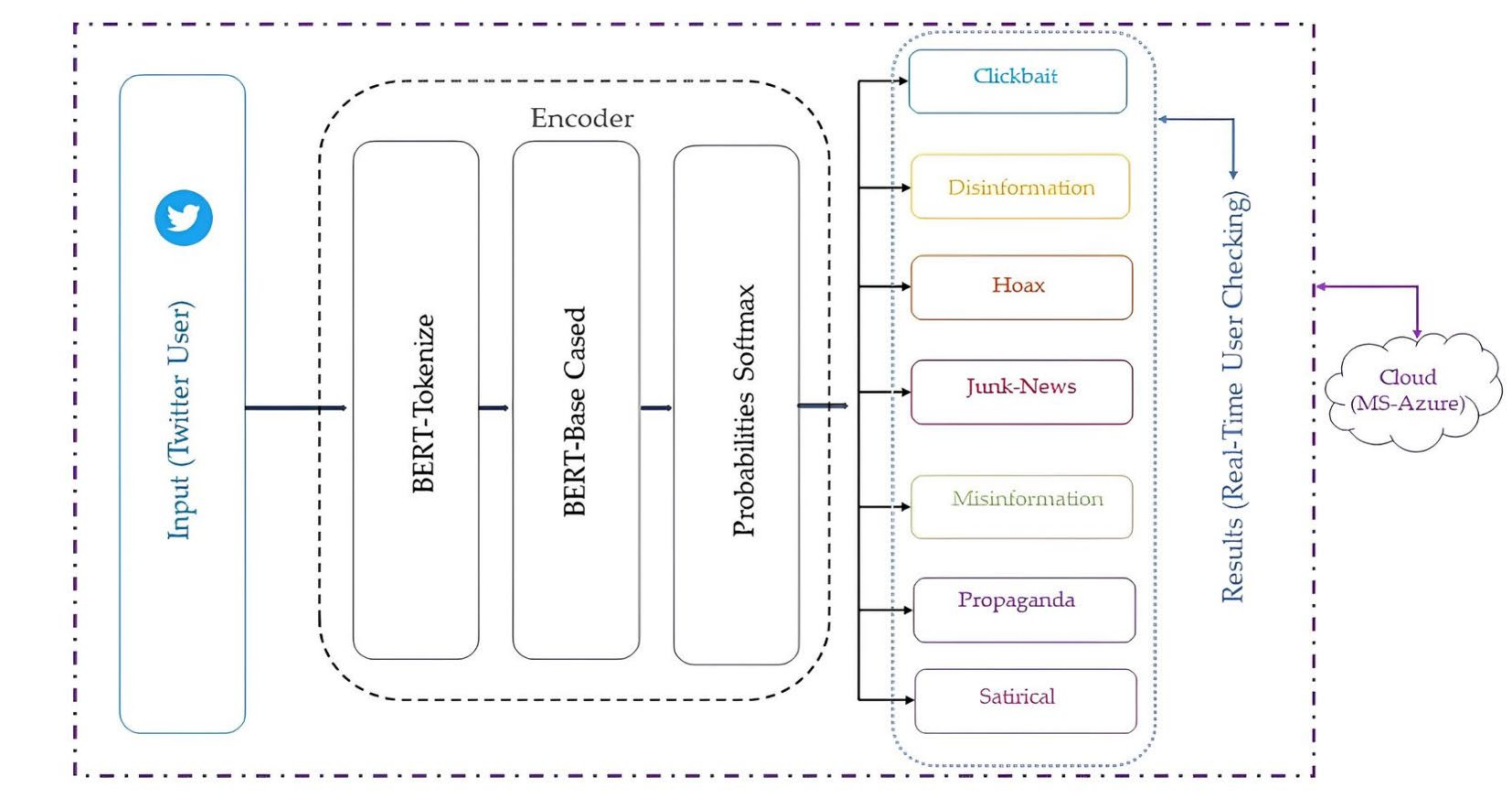
The general design of the FANDC model.

**Fig. 2:**
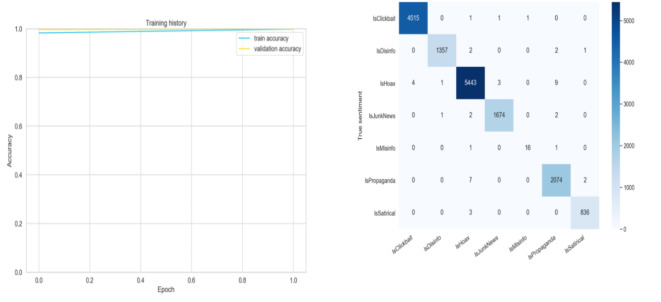
FANDC system post-training accuracy and confusion matrix of post-training.

**Fig. 3:**
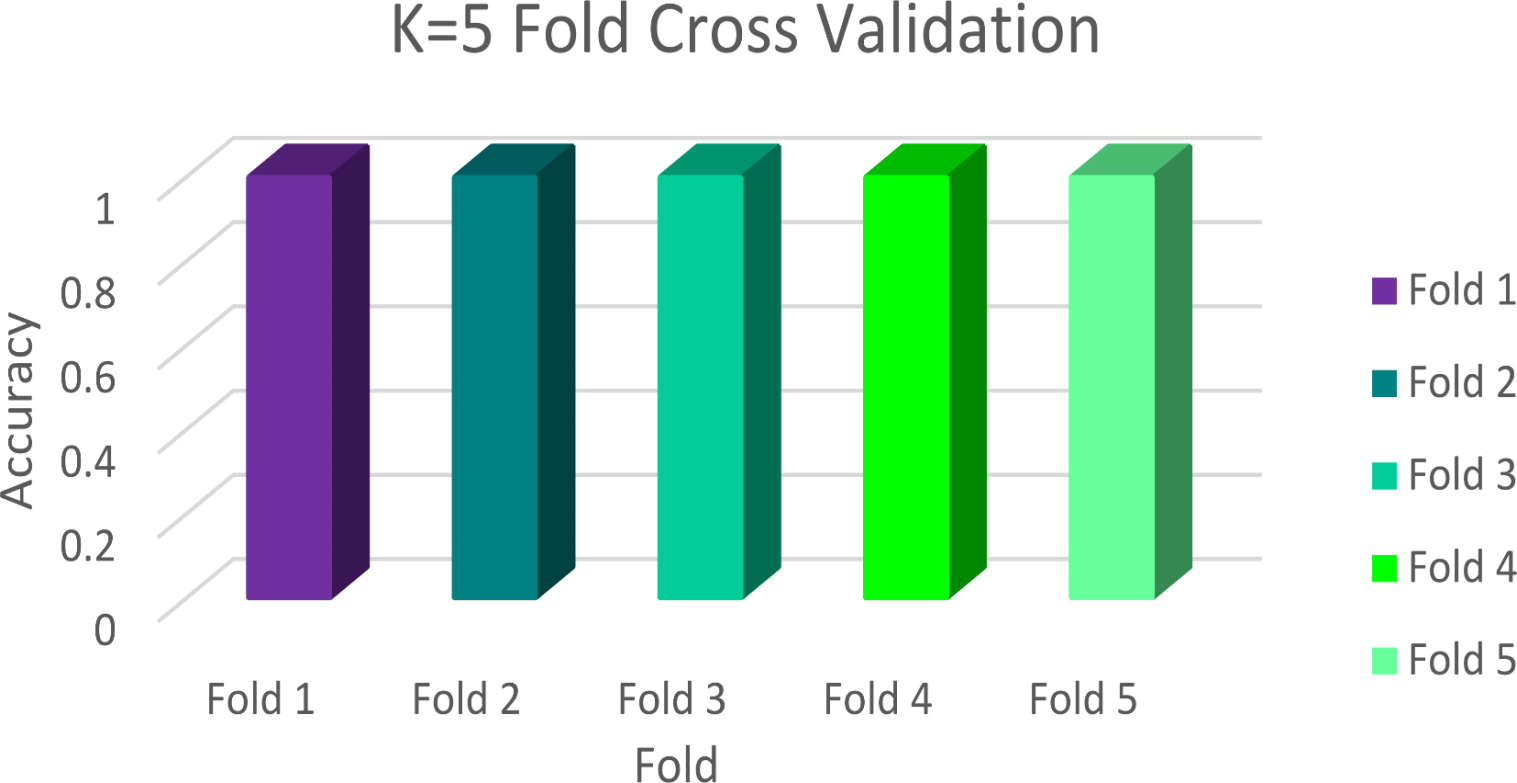
FANDC System K = 5 Fold Cross-Validation Accuracy.

The name FANDC was given to the system created to solve the problem of fake news, which increased significantly during the COVID-19 pandemic in OSNs. The general design of the FANDC system is shown in Fig. 1. In the FANDC fake news detection system, the BERT algorithm is used, which analyzes the previous and following words or similar words together instead of evaluating the words one by one. The primary goal here is to understand complex user needs better. BERT better understands how conjunctions and prepositions used in queries add meaning to a sentence. For this reason, the data set that was ready for training after the data pre-processing stage was trained with the BERT algorithm. The system is trained as 80/20 training and test data. The accuracy of the data set trained in two epochs with the BERT algorithm is 100%, as seen in Fig. 2 (a). It has been moved to the MS Azure cloud system to protect against cyber-attacks, which may be caused by the system’s online fake news detection, such as service interruption or click-bait, and to ensure data security. The confusion matrix in Fig. 2 (b) and the success rate of the classification phase after the training are shown in Table 2. After the training, click-bait, disinformation, hoax, junk news, misinformation, propaganda, and satire were trained with full accuracy, whereas propaganda was trained with 99% accuracy and misinformation with 94% accuracy. Therefore, it is evident that the system successfully carries out the training process. Additionally, the K-Fold Cross-Validation method was used to evaluate the performance of the model and measure its generalization ability, as seen in Fig. 3.

**Table 2:** The success rate of the post-training classification stage of the FANDC System.

Category	Precision	Recall	F1 Score	Support
IsClickbait	1.00	1.00	1.00	4518
IsDisinfo	1.00	1.00	1.00	1362
IsHoax	1.00	1.00	1.00	5460
IsJunkNews	1.00	1.00	1.00	1679
IsMisinfo	0.94	0.89	0.91	18
IsPropaganda	0.99	1.00	0.99	2083
IsSatirical	1.00	1.00	1.00	839
Accuracy			1.00	15,959
Macro avg	0.99	0.98	0.99	15,959
Weighted avg	1.00	1.00	1.00	15,959

**Evaluation metrics.** Various evaluation criteria have been used to measure the performance of the BERT algorithm used to detect fake news in OSNs. We examined these metrics in this section. Burkov^31^ stated the below formulas for accuracy, precision, recall, and other evaluation criteria, such as F1 scores.

1 $$\:\begin{array}{c}Accuracy=\frac{\text{T}\text{P}\:+\:\text{T}\text{N}}{\text{T}\text{P}+\:\text{F}\text{P}\:+\:\text{T}\text{N}\:+\:\text{F}\text{N}}\end{array}$$

2 $$\:\begin{array}{c}Precision=\frac{\text{T}\text{P}}{\text{T}\text{P}\:+\:\text{F}\text{P}}\:\end{array}$$

3 $$\:\begin{array}{c}Recal=\frac{\text{T}\text{P}}{\text{T}\text{P}\:+\:\text{F}\text{N}}\:\end{array}$$

4 $$\:\begin{array}{c}F1\:Score=2*\frac{\text{P}\text{r}\text{e}\text{c}\text{i}\text{s}\text{i}\text{o}\text{n}\:\text{*}\:\text{R}\text{e}\text{c}\text{a}\text{l}\text{l}\:}{\text{P}\text{r}\text{e}\text{c}\text{i}\text{s}\text{i}\text{o}\text{n}\:+\:\text{R}\text{e}\text{c}\text{a}\text{l}\text{l}}\:\end{array}$$

Where TP: True Positive, TN: True Negative, FP: False Positive, and FN: False Negative. These are all defined in the confusion matrix.

Looking at the datasets where fake news datasets are often skewed, high precision can easily be achieved by making fewer positive predictions. Therefore, recall measures the sensitivity, or proportion, of annotated fake news articles that are predicted to be fake news. F1 combines precision and recall, providing an overall predictive performance for fake news detection. The higher Precision, Recall, F1, and Accuracy value, the better the performance will be. The Receiver Operating Characteristic (ROC) curve is a graph showing the performance of a two-parameter classification model against True Positive Rate (TPR) versus False Positive Rate (FPR) at all classification thresholds. TPR and FPR are provided as follows. The area under the ROC Curve (AUC) measures the entire two-dimensional area under the ROC curve. For training and testing errors, the post-training system confusion matrix can be viewed in Fig. 2 (b). The results obtained are expressed in the macro avg line in Table 2.

5 $$\:\begin{array}{c}TPR=\frac{\text{T}\text{P}}{\text{T}\text{P}\:+\:\text{F}\text{N}}\:\end{array}$$

6 $$\:\begin{array}{c}TPR\:=\:Sensitivity\:=\:Recall\:\end{array}$$

7 $$\:\begin{array}{c}FPR=\frac{\text{F}\text{P}}{\text{F}\text{P}\:+\:\text{T}\text{N}}\:\end{array}$$

## Results

**Fig. 4:**
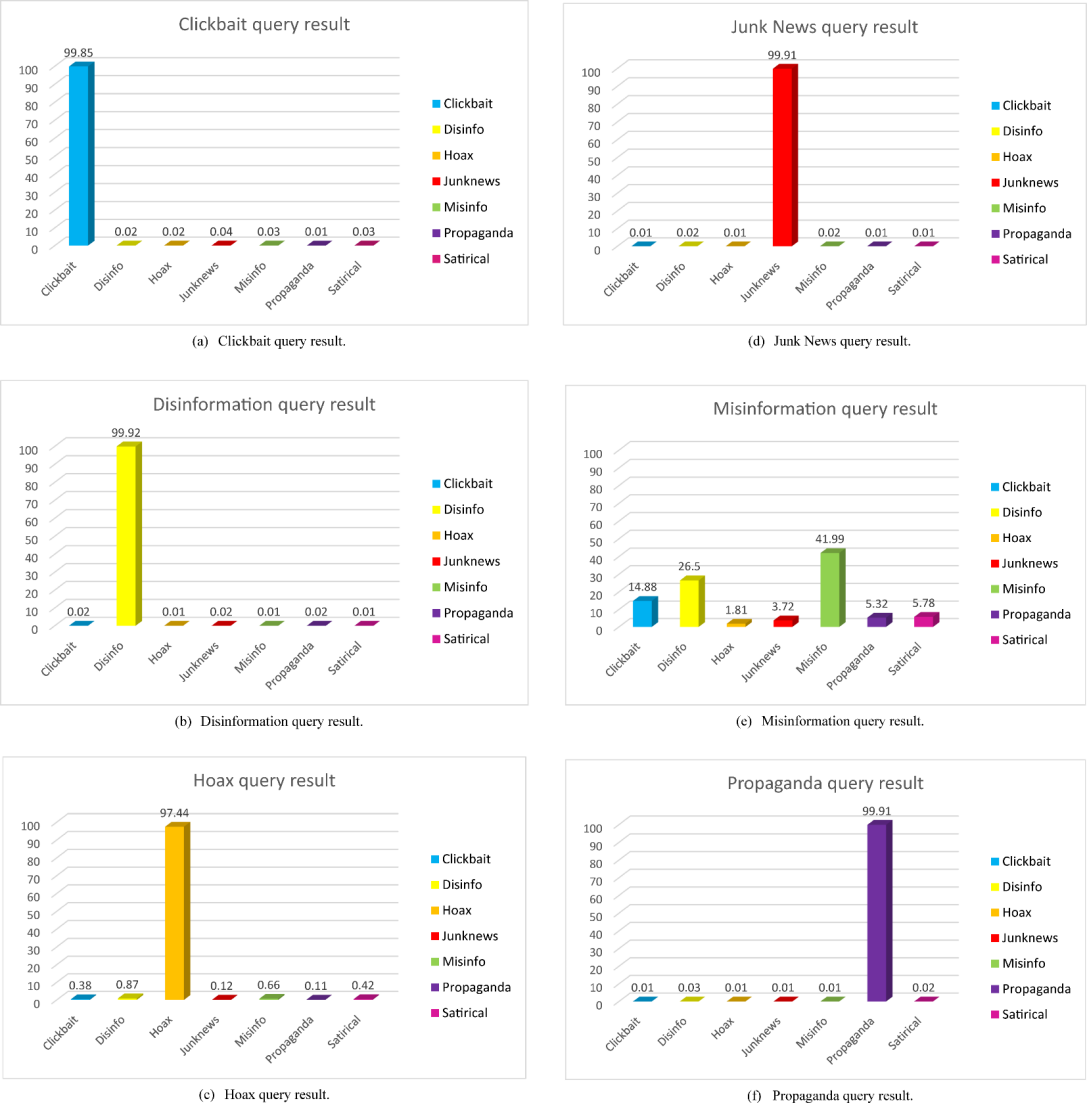

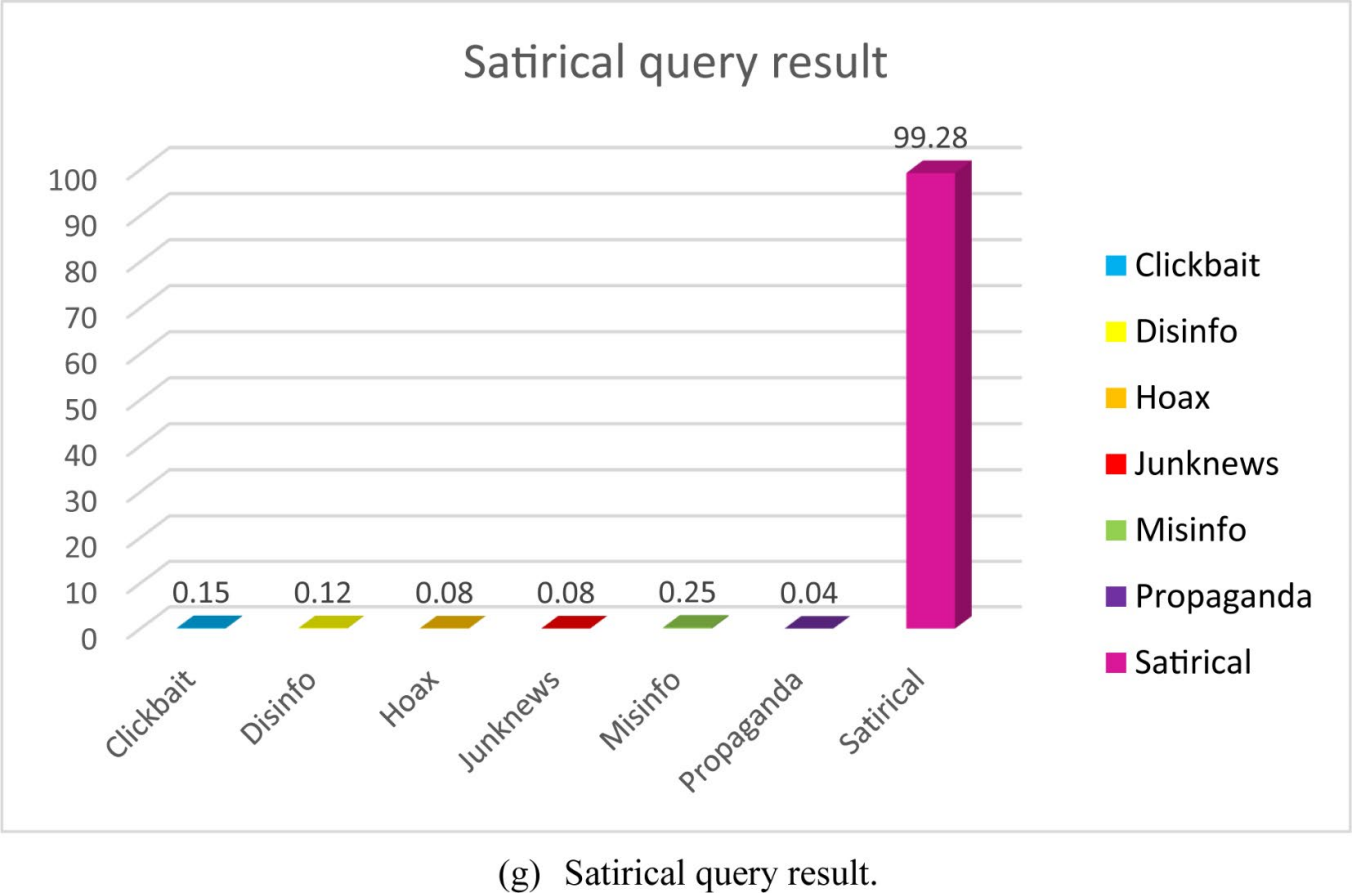
FANDC system examples of fake news analyze.

The FANDC system, which works on cloud computing and was created to detect fake news in OSNs, has been tested experimentally. At the end of the detailed analysis, the obtained results can be seen in Fig. 4 (a-g).

Figure 4 (a) shows an example of a click-bait query result. “*New research: Our latest memo on YouTube #misinfo shows that the most popular #junknews videos find their audiences through Facebook. Less than 1% %of the problematic videos shared on Facebook*,* less than 1% flagged the platform as potentially misleading. DemTech | Covid-related misinformation on YouTube: The spread of misinformation videos on social media and the effectiveness of platform policies (ox.ac.uk)*”. In this tweet, the FANDC system categorizes it as a clickbait. The webpage shortcut placed under the tweet is considered to be a click-bait trap by the system. Figure 4 (b) shows an example of the disinformation query results. The tweet text is “*Delighted to be part of this @UoYSociology event on July 26. We’re looking for proposals from any discipline on the themes of ‘Myth*,* Rumor & Misinformation’. 250 words max*,* deadline Fri 10 Jun. See all the details here*: http://bit.ly/folklore-to-fa…. *#myth #misinfo*”. When the content of a tweet is examined, the use of a word’s myth and rumor together with misinformation leads to its perception as disinformation. Figure 4 (c) shows an example of the Hoax query result. Tweet text is “*Freshman Christopher Phillips CALLS OUT Brian Stelter and CNN for being a “purveyor of disinformation” purveyor of disinformation. He points to the Russian collusion hoax Jussie Smollett*,* the smears of Justice Kavanaugh and Nick Sandmann*,* and their dismissal of Hunter Biden’s laptop*”. Figure 4 (d) shows an example of a junk news query. Tweet text is “*A Russian soldier from a chemical*,* biological*,* and nuclear protection unit picked up a source of cobalt-60 at one waste site with his bare hands*,* exposing himself to so much radiation in a few seconds that it went off the scales of a Geiger counter*”. Figure 4 (e) shows an example of a misinformation query result. Tweet text is “*NC/VA - If you are not angry about this*,* you are not paying attention! #WatchTCenergy #Disinformation Internal emails show gas pipeline firms providing NC and Virginia leaders draft letters and points to praise their projects.*https://www.huffpost.com/entry/williams-tc-energy-pipeline-projects-influence-virginia-north-carolina_n_6261e382e4b0dc52f494659e?%20utm_campaign=share_twitter&ncid=engmodushpmg00000004%E2%80%A6*via @HuffPostPol*”. Figure 4 (f) shows an example of propaganda query results. The text is “*Ontario Doctor accused of ‘disgraceful’ #c*.

*COVID-19 conduct has been suspended. Patrick Phillips spread the pandemic #misinformation and prescribed the debunked treatment #ivermectin at @lexharvs*https://thestar.com/news/canada/2022/05/03/ontario*doctor-accused-of-disgraceful-covid-conduct-gets-suspended.html. utm_source = Twitter… via @torontostar #cdnhealth #infodemic*”. In this tweet, the propaganda activities of doctors were classified as having a high success rate. Figure 4 (g) shows an example of a satirical query result. Tweet text is “*The information that the #Vatican bought Ruble for the #Russian gas is based on a #Satirical post*”. Finally, when we look at the tweets analyzed in Fig. 4 (g), it is determined that they are satirical in terms of content and are classified in this category.

The FANDC system created by prioritizing user needs within the framework of the CRISP-DM methodology, used cloud computing in a distributed and container structure to avoid cyber-attacks. In addition, the BERT algorithm, which is a proven NLP algorithm in its field, was preferred. Since the FANDC system works with big data, there was a consideration with regards to computational power as the storage capacity and processor of a computer would be insufficient. Hence, implementing the study in the cloud. The cloud-based approach ran on MS Azure Cloud Computing with 20 GB RAM, 500 GB SSD HDD and 8 Core CPU processing power.

The results obtained in this study provided feedback to OSN users in seven subcategories when detecting fake news. It enables users check suspicious posts as soon as possible. These posts, which can be evaluated with a high accuracy of 99%, provide users with great convenience and comfort in OSNs. The main focus of this study is not just fake news or its operational components, but to provide highly accurate feedback to the user in a short time span.

## Discussion

Although fake news is often viral in OSNs, users are often unprotected and helpless. Clickbait is often placed within a news story containing fake or real information to deceive OSN users. Even though Rajapaksha et al.^32^ detailed a study to detect click-bait traps by focusing on a learning model, when the results were examined, it was observed that the success rates were low despite the use of the BERT algorithm and its derivatives. The dataset used in this study consists of a labeled dataset. Again, as stated in the conclusion, it can be seen that the dataset used is insufficient in terms of volume and veracity. In this context, it is seen that the best result achieved is at the level of 90%. Although the extra-weighted method was used in the algorithms in a study by Hadi et al.^33^, the results did not reach 80%. Compared with the results of the *FANDC* system, which yielded results with 99.85% accuracy, as shown in Fig. 4 (a), the click-bait detections from other studies were at a very low level. What makes this study valuable is the volume of the dataset and meticulous preprocessing of the data.

In today’s digital world, disinformation is important as it can have an adverse effect on an affected person. Rastogi and Bansal^34^ achieved a 98% success rate for a system developed to detect disinformation. In this study an integrated approach is used, considering style-based and propagation-based features. However, when the data set is examined, it is noteworthy that it has a limited structure. The FANDC system was created by collecting large amounts of various data. Thus, more meaningful results were obtained. As seen in Fig. 4 (b), 99.92% performance was achieved.

Henry and Stattner^35^ focused on the early detection of the spread of hoaxes in their study. They achieved an accuracy rate of up to 90% in the early detection of hoax spread in OSNs within approximately 20 min. Amrullah et al.^36^ proposed a linguistic approach that could be used as the first approach for detecting hoaxes. In this approach, the speaker’s attitudes and behaviors were the focus, and the success rate could reach only 40%. Unlike other studies, Kencana et al.^37^ attempted to detect hoaxes using feed-forward and backpropagation neural network classification methods. The aforementioned study was conducted using an artificial neural network learning methodology based on deep learning algorithms. However, a success rate of 78.76% was achieved. Linge and Wicaksono^38^ conducted research on negative content on Twitter to detect hoaxes during the COVID-19 period. Classical ML algorithms were applied using the CRISP-DM methodology. The SMOTE technique was used in this study because un-balanced datasets were used. Consequently, the success rate of the algorithms was in the range–95–99%.

Research on junk news detection, a source of dissemination focused on search engines or websites (as a news source) is usually encountered. This type of fake news is commonly found in many search engines and junk news websites. For example, at the beginning of the COVID-19 epidemic, news that the disease was caused by the consumption of bat and dog meat suddenly went viral. Liotsiou et al.^39^ defined junk news as various forms of propaganda, which include but not limited to extreme ideologies, overly partisan, or conspiratorial political news and information.

Savolainen et al.^40^ conducted a study on Facebook, a commonly used OSN platform. In this study, junk news was identified by associating it with sentiment analysis within the scope of bipartisan or extremist ideas. Marchal et al.^41^ in their study on Twitter, measured the rate of junk news among all news and analyzed the results according to sentiment analysis. They found that less than 2% of the junk news was shared. The results of these studies indicate that junk news has not been examined extensively. For this reason, it was observed that the FANDC system achieved a unique result in this category with value of 99.91% accuracy as shown in Fig. 4 (d). This is because the FANDC system responds to the requests and needs of OSN users real-time. And as a result of the container structure provided by cloud computing, it is cyber-attacks resistant to a huge degree.

Misinformation is defined as false information shared without the intent to cause harm. It is another type of fake news spread through OSNs. Because OSN users often share information they believe to be true, misinformation is often difficult to detect. Therefore, due to the insufficient level of the database in the training dataset and its complex relationship with disinformation, the success of the system was only around 41.99%, as seen in Fig. 4 (e). In this context, Mulahuwaish et al.^42^ obtained 92.2% accuracy with a deep learning model in their studies by collecting Twitter data. It is remarkable that the datasets are sufficiently big and that they achieve success as a result of ten-fold training. Liu et al.^43^ on the other hand, created a data set by collecting data from WeChat and Weibo, a local microblogging site similar to Twitter used in China. They trained this dataset with 80% training and 20% testing using a tenfold cross-validation technique. The score obtained by applying classical ML algorithms was approximately 83%. Hayawi et al.^44^ created a new Twitter dataset to detect misinformation regarding the COVID-19 vaccine. They managed to create a very successful dataset with many tweets exceeding 15 M and modeled the system with 75% training and 25% testing. The results obtained using three different algorithms and different iterations reached 99% in the BERT algorithm, 84% in XGBoost in other algorithms, and approximately 99% in LSTM. Al-Rakhami and Al-Amri^45^ collected approximately 400 K tweets on Twitter and created a dataset for their study. In their study, using six classical ML algorithms, they achieved a 97% success rate using a ten-fold cross-validation technique.

Propaganda is one of the most widely used types of fake news. For this type of fake news, it is important to reach a large number of people to influence the public. Dewantara and Budi^46^ detected propaganda in online news articles by training the data in two layers, 80% training and 20% testing, using deep learning algorithms. They achieved an accuracy of 93%. However, it is noteworthy that online news sites are used instead of OSN. Polonijo et al.^47^ applied a dataset created by collecting data from online news sites using RapidMiner software, a ready-made data-mining tool that uses deep-learning algorithms. In this study, although the extent to which the dataset was separated into training and test data was not specified, a ten-fold deep learning method was used. However, results were obtained with an accuracy of up to 95%. When these studies are compared with the FANDC system, considering that our system works in real-time and on the cloud, it is seen that it responds safely to OSN users in propaganda detection with 99.9% accuracy, as seen in Fig. 4 (f).

Razali et al.^48^ study includes 32k articles trained with deep learning algorithms and collected from online news sites. The dataset was divided into 80% training data and 20% testing data. The data obtained at the end of the training were evaluated by classification using ML algorithms. Ionescu et al.^49^ and Martin et al.^50^ in their study used CamemBERT, a state-of-the-art language model for French, with the data set they created from French online news sites for the French language. They used kernel ridge regression, which is resistant to overfitting, and achieved 97.4% success in news articles. Although the system does not operate online or in real-time, it remains in the experimental stage. Compared to the FANDC system, our system is considered to be at a very good level with an accuracy rate of 99.28%, as seen in Fig. 4 (g).

It can be seen from the literature that studies focused on only one category of fake news detection. On the other hand, the FANDC system proposed in this study allows the OSN user to copy the shortcut of any tweet that suspects and wants to check from the system control bar and detect it in real time. Thus, OSN users can reach the results within seconds and see which category the suspected news is in. As a result, it is concluded that it is possible to detect fake news spread on OSNs online in real time. In addition, the fake news in question was classified into seven different categories and a more specific result was obtained contrary to the fake news general approach. In addition, the two most important factors that make the FANDC system different from other fake news detection systems are; real-time and cloud features.

## Conclusions

In recent years, despite the many benefits of OSNs, the spread of fake news on these platforms has increased daily and affected many users negatively. This has been considered a major future problem. Many systems have been developed to detect fake news spread in OSNs as previously mentioned in the discussion section. However, most of them have remained both offline and at an experimental level. It is important to note that social networking sites are naturally used by users for real-time communication purposes and users face fake news problems. Hence, the need for systems capable of real-time detection. This means there is a need for online and real-time systems. Conversely, it has been concluded that using a single method and a single category for detecting fake news cannot solve the problem. The methods explained in the related works and discussion section are both time-consuming and subjective and they do not meet end-user needs. In addition, it should avoid cyber threats and provide ubiquitous support to users. Therefore, the FANDC system was created within the framework of the CRISP-DM methodology, prioritizing user needs. To avoid cyber-attacks, cloud computing, which has a distributed and container structure, was used. In addition, the BERT algorithm, which is an NLP algorithm was also adopted Since the FANDC system works with big data, it is evaluated that the storage capacity and computational power of a computer will be insufficient, hence, the need for a cloud-based approach over running om a local computer and it was run on MS Azure Cloud with 20 GB RAM, 500 GB SSD HDD, and 8 Core CPU processing power. As a result, the FANDC system, which focuses on fake news for user needs achieved a 99% accuracy, 98% recall is 98%, and 99% F1-score. In addition to the FANDC system detecting fake news with 99% accuracy, it can also classify them into seven subcategories. However, this study was limited to the mentioned system features and data from Twitter, one of the OSNs.

Consequently, OSN users will be able to easily solve the fake news detection problem with the developed system FANDC, and users will be able to confirm their suspected news in OSNs in a total of seven categories, such as junk news, satire, disinformation, misinformation, propaganda, deception, rumor, and click-bait. We hope that OSN users will be protected from the negativity that may arise from fake news viral in OSNs by the developed system FANDC. It is planned that the FANDC System will provide online service to OSN users in the future by being developed with large language models such as OLMo, ChatGPT and GEMINI, which are in constant development in the field of NLP.

## Data Availability

The data set used in this research was obtained using the Twitter API on “GitHub.com“^30^ and can be downloaded from “https://github.com/echen102/COVID-19-TweetIDs”.
